# Adherence to a Mediterranean-style diet and incident fractures: pooled analysis of observational evidence

**DOI:** 10.1007/s00394-017-1432-0

**Published:** 2017-03-22

**Authors:** Setor K. Kunutsor, Jari A. Laukkanen, Michael R. Whitehouse, Ashley W. Blom

**Affiliations:** 1 Musculoskeletal Research Unit, School of Clinical Sciences, University of Bristol, Learning and Research Building (Level 1), Southmead Hospital, Southmead Road, Bristol, BS10 5NB UK; 2 Institute of Public Health and Clinical Nutrition, University of Eastern Finland, Kuopio, Finland; 3 Central Finland Central Hospital, Jyväskylä, Finland

**Keywords:** Mediterranean diet, Bone, Fractures, Nutrition

## Abstract

**Purpose:**

The Mediterranean diet is associated with decreased morbidity and mortality from various chronic diseases. Adherence to a Mediterranean-style diet has been suggested to have protective effects on bone health and decreases the incidence of bone fractures, but the evidence is not clear. We conducted a systematic review and meta-analysis of available observational studies to quantify the association between adherence to a Mediterranean-style diet, as assessed by the Mediterranean Diet Score (MDS), and the risk of fractures in the general population.

**Methods:**

Relevant studies were identified in a literature search of MEDLINE, EMBASE, Web of Science, and reference lists of relevant studies to October 2016. Relative risks (RRS) with 95% confidence intervals (CIs) were aggregated using random-effects models.

**Results:**

Five observational studies with data on 353,076 non-overlapping participants and 33,576 total fractures (including 6,881 hip fractures) were included. The pooled fully adjusted RR (95% CI) for hip fractures per 2-point increment in adherence to the MDS was 0.82 (0.71–0.96). Adherence to the MDS was not associated with the risk of any or total fractures based on pooled analysis of only two studies.

**Conclusion:**

Limited observational evidence supports a beneficial effect of adherence to a Mediterranean-style diet on the incidence of hip fractures. Well-designed intervention studies are needed to elucidate the relationship between adherence to a Mediterranean-style diet and the risk of adverse bone health outcomes such as fractures.

## Introduction

The traditional Mediterranean diet which is characterized by high consumption of olive oil, fruits, vegetables, nuts, legumes, and cereals; moderate consumption of fish, poultry, and alcohol; and low consumption of processed food, red meat, dairy, and sweets [1] has been suggested as the optimal diet for the primary prevention of various non-communicable diseases. To assess the degree of adherence to a Mediterranean diet, the Mediterranean Diet Index was developed [2]; this index and its modification [the alternate or modified Mediterranean Diet Score (MDS)], which can be applied to non-Mediterranean populations, have been shown to have beneficial effects on health outcomes [2]. Adherence to a Mediterranean-style diet has been suggested to have beneficial effects on bone health [3]. To our knowledge, there is no published evidence of a clinical trial which shows a beneficial effect of a Mediterranean-style diet on adverse bone health outcomes such as fractures and osteoporosis. However, a limited number of epidemiological observational studies have suggested a protective effect of a high MDS on the risk of fractures, but the available evidence to date is inconsistent and inconclusive [4, 5]. We aimed to clarify the existing evidence by pooling data from available published observational cohort studies which have examined the associations between adherence to a Mediterranean-style diet and the risk of fractures in general population settings.

## Methods

This review was conducted in line with PRISMA and MOOSE guidelines (Appendices 1, 2). We searched MEDLINE, EMBASE, and Web of Science electronic databases up to October 17, 2016, using free and medical subject headings and combination of key words related to “Mediterranean diet” and “fracture.” There were no restrictions on language. Bibliographies of all retrieved articles and other relevant publications, including reviews, were manually scanned for citations missed by the electronic search. Details on our search strategy are presented in Appendix 3. Summary measures were presented as relative risks (RRs) with 95% confidence intervals (CIs). To ensure consistency in the analysis, relevant risk estimates from each study were standardized to compare a two-point increment in the Mediterranean Diet Score (MDS), using methods previously described (Appendix 4). Where studies reported differing degrees of adjustment, the multivariable-adjusted estimate that included adjustment for fracture risk factors was used. Summary RRs were calculated by pooling study-specific estimates using a random effects model. Statistical heterogeneity across studies was quantified using the Cochrane *χ* ^2^ statistic and the *I* ^2^ statistic. All analyses were performed using STATA release 14 (StataCorp LP, College Station, TX, USA) software.

## Results

**Fig. 1:**
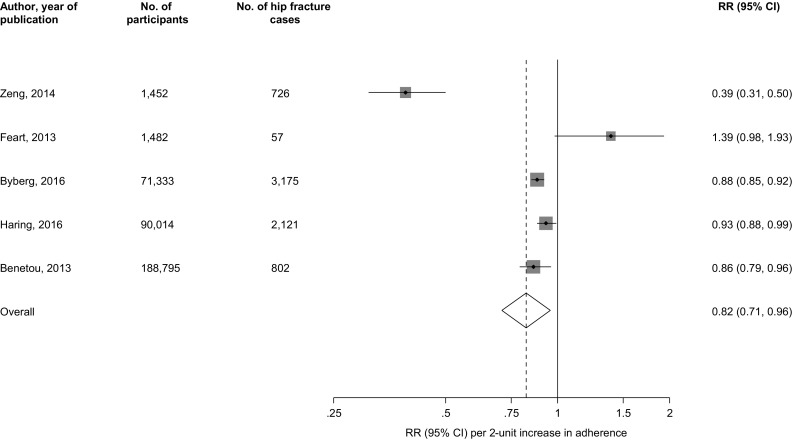
Association between adherence to a Mediterranean-style diet and risk of hip fractures in observational cohort studies. *CI* confidence interval (*bars*); *RR* relative risk; the RRs for fractures are per two-point increment in adherence to the Mediterranean Diet Score

The search strategy identified 174 potentially relevant articles. After the initial screening of titles and abstracts, 12 articles remained for further evaluation. Following detailed evaluation which included full text reviews, 7 articles were excluded. Five observational (four prospective cohort and one case–control) studies based in general populations were found to be eligible (Appendix 5). Eligible studies were published between 2013 and 2017. The studies involved 353,076 individuals aged 35–80 years at baseline, with 33,576 fractures (including 6881 hip fractures), collected over median or average follow-up periods that ranged from 8 to 15.9 years (Table 1) [4–8]. All five studies reported on hip fractures, with two of them additionally reporting on any or total fractures [4, 5]. Only one study reported on other bone health outcomes such as bone mineral density (BMD) and muscle mass [4]. Three studies were based in Europe, one in North America (USA), and one in Asia (China). The RR for hip fractures per two-point increment in adherence to the MDS, typically adjusted for several conventional risk factors, was 0.82 (95% CI 0.71–0.96) (Fig. 1). There was evidence of substantial heterogeneity (>70%) among the included studies. Egger’s regression test showed no statistical evidence of publication bias (*P* = 0.603). When analysis was restricted to the two studies that reported on any or total fractures (comprising 91,496 individuals and 28,873 fractures), the corresponding pooled RR was 1.00 (95% CI 0.99–1.02). The absolute risk reduction (ARR) of hip fractures associated with a two-point increment in adherence to the MDS was 0.18%, which translates into a number needed to treat (NNT) of 556 (95% CI 345–2500) to prevent one hip fracture.

**Table 1:** Characteristics of published observational studies evaluating associations between adherence to a Mediterranean-style diet and incident fractures

Lead author, publication year [Reference]	Name of study or source of participants	Location of study	Year(s) of baseline survey	Baseline mean age (age range), years	% male	Mean or median duration of follow-up (years)	Total no. of participants	No. of cases	Primary outcome	Other bone health outcomes	Covariates adjusted for	Exposure	Mean (SD) or median (range) for MDS
Benetou, 2013 [6]	EPIC	Multi-European	1992–2000	48.6 (35–70)	25.9	9.0.0	188,795	802	Hip fractures	NA	Age, sex, education, smoking status, BMI, height, physical activity, total energy intake, history of diabetes, history of CVD, history of cancer, history of fracture, and country	Modified Mediterranean Diet Score	NR
Feart, 2013 [5]	Three-City Study	France	2001–2002	47.8 (≥67)	37.1	8.0	1482	155	Hip and any fractures (hip, vertebral, or wrist fractures)	NA	Age, gender, physical activity, total energy intake, additional adjustment for educational level, marital status, BMI, self-reported osteoporosis, osteoporosis treatment, calcium and/or vitamin D treatment	Mediterranean Diet Score	4.38 (1.68)
Zeng, 2014 [7]	Hip fracture patients and community-sourced controls	China	2009–2013	71.0 (55–80)	24.4	NA	1452	726	Hip fractures	NA	Age, BMI, education, marital status, occupation, household income, house orientation, smoking status, tea drinking, family history of fractures, calcium supplement user, multivitamin user, physical activity, and daily energy intake	Alternate Mediterranean Diet Score	3 (0–7) in cases 4 (0–8) in controls
Haring, 2016 [4]	WHI Observational Study	USA	1993–1998	(50–79)	0.0	15.9	90,014	28,718	Hip and total fractures (all fractures except toe, finger, sternum, and clavicle fractures)	BMD and lean body mass index	Age, race/ethnicity, BMI, smoking status, physical activity, self-reported health, DM, history of fracture at 55 years or older, physical function score, number of chronic medical conditions, number of psychoactive medications, and use of hormone therapy, bisphosphonates, calcitonin, and selective estrogen receptor modulators	Alternate Mediterranean Diet Score	NR
Byberg, 2016 [8]	COSM and SMC	Sweden	1997	60.0 (NR)	53.1	15.0	71,333	3175	Hip fractures	NA	Age, sex, BMI, height, DM, smoking status, physical exercise, educational level, living alone, total energy intake, energy adjusted intake of calcium, vitamin D and retinol, use of supplements containing calcium or vitamin D, and Charlson’s weighted comorbidity index	Modified Mediterranean Diet Score	NR

*BMD* bone mineral density, *BMI* body mass index, *COSM* Cohort of Swedish Men, *CVD* cardiovascular disease, *DM* diabetes mellitus, *EPIC* European Prospective Investigation into Cancer, *MDS* Mediterranean diet score, *NA* not applicable, *NR* not reported, *SMC* Swedish Mammography Cohort, *WHI* Womens Health Initiative

## Discussion

Emerging evidence from observational cohort studies published only within the last 4 years and involving apparently healthy participants indicates that increasing adherence to a Mediterranean-style diet is associated with lower risk of hip fractures; however, the risk reduction is low. Our results add to the existing evidence that adherence to a Mediterranean diet is protective of adverse health outcomes such as cardiovascular disease, cancer, and neurodegenerative diseases [9], as well as all-cause mortality [9]. Although a limited number of studies have suggested a beneficial effect of the Mediterranean-style diet on the incidence of bone fractures, the results have mostly been inconsistent. By pooling the few published studies on the topic, we have shown that increased adherence to a Mediterranean-style diet is associated with reduced incidence of hip fractures among general population settings. However, pooled analysis of the only two published studies reporting on any or total fractures showed no statistically significant evidence of an association. Feart and colleagues in analysis of a cohort of French elderly people showed no evidence of associations of adherence to a Mediterranean-style diet with risk of any as well as hip fractures; however, their analysis was hampered by the small size [5]. In a recent post hoc analysis of over 90,000 participants recruited in the Women’s Health Initiative (WHI) observational study, Haring and colleagues showed that higher adherence to a Mediterranean diet was associated with a reduced risk of hip fractures but not total fractures [4]. In the WHI study, the lack of an association between adherence to a Mediterranean diet and total fracture risk was potentially attributed to the wide variation of fracture types included in the analyses. Outcome events on any fractures from these two studies were self-reported, which increased the likelihood of misclassification bias. It has been suggested that the protective effects of the Mediterranean diet on fracture risk may be via its effect on BMD and muscle mass [4, 10]. However, in the WHI study, the authors found no significant changes in BMD and lean body mass over time with adherence to a Mediterranean diet [4].

The Mediterranean diet has been suggested to have a beneficial effect on bone health, and this has been attributed to the antioxidant, anti-inflammatory, and alkalinising properties of the naturally occurring bioactive compounds within this diet [11]. Although the bone protective effects of the Mediterranean-style diet are attributed to the combination of the individual components of the diet, it has been suggested that key components of this diet may be responsible for its protective effect on bone mineral density (i.e., osteoporosis) and fracture occurrence [6]. Our findings have potential clinical implications, as hip fractures (particularly osteoporotic fractures) are one of the leading worldwide causes of disability and morbidity, especially in elderly patients, and increase the burden on health systems. The prevention of fractures is therefore of public health importance. Our ARR estimate of 0.18% as suggested by the pooled analysis translates to about 5,004 people having a two-point increment in adherence to the MDS to prevent one hip fracture in a year. However, this estimate assumes that the effect of adherence to the MDS is constant over time and with hip fracture events occurring at a constant rate over time [12]. The ARR estimate does not seem encouraging; however, it is well known that adherence to the Mediterranean-style diet has beneficial effects on several outcomes. Although bone mass and the risk of fractures are determined by a combination of aging, heritability, mechanical (such as physical activity), and hormonal factors, nutrition plays an important role in bone health. The evidence of a protective effect of nutrition on bone health has mostly been based on specific dietary factors such as calcium, vitamin D, or other isolated nutrients [13, 14], though the role of proteins remains controversial [15, 16]. The current findings suggest that the combined beneficial effects of the individual dietary components which make up the Mediterranean-style diet may represent an appropriate and feasible dietary intervention for the prevention of bone fractures, rather than the promotion of isolated nutrients. Although residual confounding may have explained part of the findings, at least adherence to a Mediterranean diet did not have a harmful effect on bone health. Given that the Mediterranean diet does not emphasize nutrients that have been suggested to have a beneficial effect on bone health such as calcium or protein intake, it is assuring to see that beyond other well established benefits of a Mediterranean diet; there are no detrimental effects of this diet on bone health.

To our knowledge, this is the first study to evaluate relevant studies that have assessed associations between adherence to a Mediterranean-style diet and the risk of fractures using a systematic meta-analytic approach. We were able to harmonize data from the limited studies conducted on the topic to perform a quantitative analysis, thereby obtaining reliable estimates of the nature and magnitude of the association between adherence to a Mediterranean-style diet and the risk of fractures. There were no relevant clinical trials published on this specific topic; therefore our review was based on only observational evidence. Substantial heterogeneity was observed between contributing studies and which could not be explored because of the limited number of studies. We acknowledge the country-specific characteristics of the Mediterranean dietary pattern, which may explain the different study-specific effect sizes as well as substantial heterogeneity among studies. Indeed, it has been shown that different dietary patterns even exist among Mediterranean countries [17]. Although each eligible study adjusted for a comprehensive panel of confounders including vitamin D, history of fracture, and physical activity (which are major risk factors for hip fracture), the study estimates are still prone to residual confounding because of the observational nature of the study designs. For example, studies did not take into account the mechanisms of fracture occurrence such as falls in their analysis; falls are known to influence hip fracture risk beyond BMD [18]. In addition, adherence to a Mediterranean diet may rather reflect a healthier lifestyle which was not completely captured by confounders that were included in the various analyses. Inadequate data on sex-specific estimates precluded assessment of the associations in males and females separately. However, limited data from the individual studies suggest that the protective effect of adherence to a Mediterranean-style diet on hip fractures is more evident in men compared with women. Even though we detected no evidence of publication bias, we were unable to adequately explore for this given that tests for publication bias are unlikely to be useful for analysis involving limited number of studies. Finally, our NNT estimate was calculated from an observational design; ideally, it should have been based on findings from a randomized controlled trial. The findings should therefore be interpreted with caution given these limitations.

In conclusion, available evidence suggests a beneficial effect of adherence to a Mediterranean-style diet on the incidence of hip fractures; however, the pooled risk reduction is low. This review also highlights the limited evidence on the topic in the existing literature and therefore the need for robust well-designed intervention studies to elucidate the relationship between adherence to a Mediterranean-style diet and the risk of adverse bone health outcomes such as fractures and osteoporosis.

### Author contribution

SKK, JAL, MRW, and AWB conducted and designed research; SKK analyzed and interpreted data. SKK and JAL contributed to data acquisition. SKK wrote the paper, and JAL, MRW, and AWB contributed to the interpretation of data. All authors read and approved the final manuscript.

## Appendix 1: PRISMA checklist

**Table:**

Section/topic	Item No	Checklist item	Reported on page No
Title			
Title	1	Identify the report as a systematic review, meta-analysis, or both	Title
Abstract			
Structured summary	2	Provide a structured summary including, as applicable, background, objectives, data sources, study eligibility criteria, participants, interventions, study appraisal, synthesis methods, results, limitations, conclusions and implications of key findings, and systematic review registration number	Introduction
Introduction			
Rationale	3	Describe the rationale for the review in the context of what is already known	Introduction
Objectives	4	Provide an explicit statement of questions being addressed with reference to participants, interventions, comparisons, outcomes, and study design (PICOS)	Introduction
Methods			
Protocol and registration	5	Indicate if a review protocol exists, if and where it can be accessed (such as web address), and, if available, provide registration information including registration number	Not applicable
Eligibility criteria	6	Specify study characteristics (such as PICOS and length of follow-up) and report characteristics (such as years considered, language, and publication status) used as criteria for eligibility, giving rationale	Methods
Information sources	7	Describe all information sources (such as databases with dates of coverage and contact with study authors to identify additional studies) in the search and date last searched	Methods
Search	8	Present full electronic search strategy for at least one database, including any limits used, such that it could be repeated	Appendix 3
Study selection	9	State the process for selecting studies (that is, screening, eligibility, included in systematic review, and, if applicable, included in the meta-analysis)	Methods
Data collection process	10	Describe method of data extraction from reports (such as piloted forms, independently, in duplicate) and any processes for obtaining and confirming data from investigators	Methods
Data items	11	List and define all variables for which data were sought (such as PICOS and funding sources) and any assumptions and simplifications made	Methods
Risk of bias in individual studies	12	Describe methods used for assessing risk of bias of individual studies (including specification of whether this was done at the study or outcome level), and how this information is to be used in any data synthesis	Methods
Summary measures	13	State the principal summary measures (such as risk ratio and difference in means)	Methods
Synthesis of results	14	Describe the methods of handling data and combining results of studies, if done, including measures of consistency (such as I^2^ statistic) for each meta-analysis	Methods
Risk of bias across studies	15	Specify any assessment of risk of bias that may affect the cumulative evidence (such as publication bias and selective reporting within studies)	Methods
Additional analyses	16	Describe methods of additional analyses (such as sensitivity or subgroup analyses and meta-regression), if done, indicating which were pre-specified	Not applicable
Results			
Study selection	17	Give numbers of studies screened, assessed for eligibility, and included in the review, with reasons for exclusions at each stage, ideally with a flow diagram	Results and Figure
Study characteristics	18	For each study, the present characteristics for which data were extracted (such as study size, PICOS, and follow-up period) and provide the citations	Table
Risk of bias within studies	19	Present data on risk of bias of each study and, if available, any outcome-level assessment (see item 12)	Table
Results of individual studies	20	For all outcomes considered (benefits or harms), present for each study (a) simple summary data for each intervention group and (b) effect estimates and confidence intervals, ideally with a forest plot	Figure
Synthesis of results	21	Present results of each meta-analysis done, including confidence intervals and measures of consistency	Figure
Risk of bias across studies	22	Present results of any assessment of risk of bias across studies (see item 15)	Not applicable
Additional analysis	23	Give results of additional analyses, if done (such as sensitivity or subgroup analyses, meta-regression) (see item 16)	Not applicable
Discussion			
Summary of evidence	24	Summarize the main findings including the strength of evidence for each main outcome; consider their relevance to key groups (such as health care providers, users, and policy makers)	Discussion
Limitations	25	Discuss limitations at study and outcome level (such as risk of bias), and at review level (such as incomplete retrieval of identified research and reporting bias)	Discussion
Conclusions	26	Provide a general interpretation of the results in the context of other evidence and implications for future research	Discussion
Funding			
Funding	27	Describe sources of funding for the systematic review and other support (such as supply of data) and role of funders for the systematic review	Discussion

## Appendix 2: MOOSE checklist

Adherence to a Mediterranean-style diet and incident fractures: pooled analysis of observational evidence

**Table:**

Criteria		Brief description of how the criteria were handled in the review
Reporting of background		
√	Problem definition	The Mediterranean diet is associated with decreased morbidity and mortality from various chronic diseases. Adherence to a Mediterranean-style diet has been suggested to have protective effects on bone health and decreases the incidence of bone fractures, but the evidence is not clear. We conducted a systematic review and meta-analysis of available observational studies to quantify the association between adherence to a Mediterranean-style diet, as assessed by the Mediterranean Diet Score (MDS), and the risk of fractures in the general population
√	Hypothesis statement	Adherence to a Mediterranean-style diet is associated with decreased risk of fractures
√	Description of study outcomes	Any fractures
√	Type of exposure	Adherence to a Mediterranean-style diet
√	Type of study designs used	Longitudinal studies (prospective or retrospective case–control, prospective cohort, retrospective cohort, case-cohort, nested case–control, or clinical trials)
√	Study population	Participants based in general populations in whom adherence to a Mediterranean-style diet has been assessed and have been followed-up for fracture outcomes
Reporting of search strategy should include		
√	Qualifications of searchers	Setor Kunutsor, PhD; Jari Laukkanen, PhD
√	Search strategy, including time period included in the synthesis and keywords	Time period: from inception to October, 2016 The detailed search strategy can be found in Appendix 3
√	Databases and registries searched	MEDLINE, EMBASE, and Web of Science, and Cochrane databases
√	Search software used, name and version, including special features	OvidSP was used to search EMBASE and MEDLINE EndNote used to manage references
√	Use of hand searching	We searched bibliographies of retrieved papers
√	List of citations located and those excluded, including justifications	Details of the literature search process are outlined in the flow chart in Appendix 5
√	Method of addressing articles published in languages other than English	We placed no restrictions on language
√	Method of handling abstracts and unpublished studies	Not applicable
√	Description of any contact with authors	Not applicable
Reporting of methods should include		
√	Description of relevance or appropriateness of studies assembled for assessing the hypothesis to be tested	Detailed inclusion and exclusion criteria are described in the “Methods” section
√	Rationale for the selection and coding of data	Data extracted from each of the studies were relevant to the population characteristics, study design, exposure, and outcome
√	Assessment of confounding	We assessed confounding by ranking individual studies on the basis of different adjustment levels and performed subgroup analyses to evaluate differences in the overall estimates according to levels of adjustment
√	Assessment of study quality, including blinding of quality assessors; stratification or regression on possible predictors of study results	Study quality was assessed based on the nine-star Newcastle–Ottawa Scale using pre-defined criteria namely: population representativeness, comparability (adjustment of confounders), ascertainment of outcome
√	Assessment of heterogeneity	Heterogeneity of the studies was quantified with I^2^ statistic that provides the relative amount of variance of the summary effect due to the between-study heterogeneity and explored using meta-regression and stratified analyses
√	Description of statistical methods in sufficient detail to be replicated	Description of methods of meta-analyses, sensitivity analyses, meta-regression, and assessment of publication bias are detailed in the methods. We performed random effects meta-analysis with Stata 14
√	Provision of appropriate tables and graphics	Table and Figure
Reporting of results should include		
√	Graph summarizing individual study estimates and overall estimate	Figure
√	Table giving descriptive information for each study included	Table
√	Results of sensitivity testing	Sensitivity analysis was conducted to assess the influence of some large studies and low-quality studies on the pooled estimate. This was done by omitting such studies and calculating a pooled estimate for the remainder of the studies
√	Indication of statistical uncertainty of findings	95% confidence intervals were presented with all summary estimates, I^2^ values and results of sensitivity analyses
Reporting of discussion should include		
√	Quantitative assessment of bias	Sensitivity analyses indicate heterogeneity in strengths of the association due to most common biases in observational studies. The systematic review is limited in scope, as it involves limited number of studies
√	Justification for exclusion	All studies were excluded based on the pre-defined inclusion criteria in methods section
√	Assessment of quality of included studies	Brief discussion included in ‘Methods’ section
Reporting of conclusions should include		
√	Consideration of alternative explanations for observed results	Discussion
√	Generalization of the conclusions	Discussed in the context of the results
√	Guidelines for future research	We recommend well-designed observational studies as well as clinical trials
√	Disclosure of funding source	Not applicable

## **Appendix 3**: Literature search strategy

Relevant studies, published from inception to October 17, 2016 (date last searched), were identified through electronic searches not limited to the English language using MEDLINE, EMBASE, and Web of Science, databases. Electronic searches were supplemented by scanning reference lists of articles identified for all relevant studies (including review articles), by hand searching of relevant journals and by correspondence with study investigators. The computer-based searches combined search terms related to Mediterranean diet and fracture without language restriction

Exp Diet, Mediterranean/or Mediterranean.mp. (31745)

Fracture.mp. (167252)

1 and 2 (57)

Limit 3 to humans (49)

Each part was specifically translated for searching the other databases (EMBASE, Web of Science, and Cochrane databases).

## **Appendix 4**: Risk conversion method

To enable a consistent approach to the meta-analysis and enhance interpretation of findings, risk estimates for the association of adherence to the Mediterranean Diet Score (MDS) and fracture risk that were often differently reported by each study [e.g., per unit change, quintiles, quartiles, or other groupings] were transformed to compare a two-point increment in the MDS, using standard statistical methods.^1,2^ This method requires that the number of cases, person-years of follow-up or non-cases, and the risk estimates with the variance estimates for at least three quantitative categories of the MDS are known. The median or mean level of MDS for each category was assigned to each corresponding risk estimate. If data were not available, we estimated the median using the midpoint of each category. When the highest or lowest category was open, we assumed it to be the same amplitude as the adjacent category. A dose–response analysis was then performed using the method of generalized least squares for trend estimation of summarized dose–response data,^3^ which converts the estimates to a per unit increase. For majority of studies that reported risk estimates per one-point increment in the MDS, these were converted to a two-point increment.

**Table:**

References
Chêne, G and Thompson, SG. Methods for Summarizing the Risk Associations of Quantitative Variables in Epidemiologic Studies in a Consistent Form. American Journal of Epidemiology. 1996;144:610–621
Greenland, S and Longnecker, MP. Methods for trend estimation from summarized dose–response data, with applications to meta-analysis. American Journal of Epidemiology. 1992;135:1301–1309
Orsini N, Bellocco R, Greenland S. Generalized least squares for trend estimation of summarized dose–response data. Stata Journal. 2006; 6:40–57
Kunutsor SK, Apekey TA, Walley J. Liver aminotransferases and risk of incident type 2 diabetes: a systematic review and meta-analysis. Am J Epidemiol. 2013; 178 (2): 159 − 17
